# Cyanoremediation and phyconanotechnology: cyanobacteria for metal biosorption toward a circular economy

**DOI:** 10.3389/fmicb.2023.1166612

**Published:** 2023-05-30

**Authors:** Matilde Ciani, Alessandra Adessi

**Affiliations:** Department of Agriculture, Food, Environment and Forestry (DAGRI), University of Florence, Florence, Italy

**Keywords:** cyanobacteria, bioremediation, heavy metal biosorption, metal nanoparticles, sustainability

## Abstract

Cyanobacteria are widespread phototrophic microorganisms that represent a promising biotechnological tool to satisfy current sustainability and circularity requirements. They are potential bio-factories of a wide range of compounds that can be exploited in several fields including bioremediation and nanotechnology sectors. This article aims to illustrate the most recent trends in the use of cyanobacteria for the bioremoval (i.e., cyanoremediation) of heavy metals and metal recovery and reuse. Heavy metal biosorption by cyanobacteria can be combined with the consecutive valorization of the obtained metal-organic materials to get added-value compounds, including metal nanoparticles, opening the field of phyconanotechnology. It is thus possible that the use of combined approaches could increase the environmental and economic feasibility of cyanobacteria-based processes, promoting the transition toward a circular economy.

## 1. Introduction

In recent years environmental pollution has become one of the main concerns troubling societies due to its detrimental effect on human health, ecosystems, and the ways it can affect ecological balance and resource availability. Human activity is incessantly contributing to increasing concentrations of different polluting compounds in the environment. Within organic and inorganic pollutants, heavy metals, including cadmium, chromium, copper, lead, mercury, nickel, selenium, and zinc, are commonly found in all ecosystems. Despite some metals being essential for living organisms, all metals become toxic at high concentrations (US EPA, 2015). Their non-biodegradable nature causes their accumulation in the environment as well as their magnification through the food chain with a mutagenic and/or carcinogenic risk for humans.

Metal-based industries, such as those that involve mining and electroplating activity, produce effluents with high heavy metal content (Briffa et al., 2020), which become the main cause of redistribution and concentration of the metals in water, soil, and air ecosystems and generating a danger to aquatic life, water availability for rural and urban areas, and human beings. Thus, stringent regulations, efficient technologies, and long-term sustainable strategies are essential for reducing the accumulation of heavy metals in the environment. In addition, the possibility of recovering and reusing these compounds may represent an economic opportunity for the industry and could also sustain the development of “end of waste” processes through the implementation of a circular economy approach.

Cyanobacteria are cosmopolitan photoautotrophic bacteria that represent the largest and widest group of microorganisms. Their metabolic diversity represents a rich source of biotechnological instruments for sustainable development (Mona et al., 2020; Priyanka et al., 2020). Their ability to survive in extreme conditions, comprising environments containing pesticides, petroleum by-products, radioactive compounds, crude oils, xenobiotics, and heavy metals, has drawn increasing interest from the scientific community, shedding light on the cellular mechanisms involved as well as their possible exploitation as a clean green technology for degradation or detoxification of contaminants. Many studies have been carried out for soil and water bioremediation adopting cyanobacteria. Such a process is also named cyanoremediation (Mona et al., 2020; Rueda et al., 2020; Dutta et al., 2022; Zanganeh et al., 2022). The possibility of cultivating cyanobacteria on a large scale in large ponds, coupled with the ability to fix carbon dioxide as well as atmospheric nitrogen (for some genera), makes them self-sufficient in terms of adaptability, growth, and maintenance in controlled or contaminated environments (Gehlot et al., 2022). Furthermore, the use of cyanobacteria for bioremediation is enhanced also by their ability to tolerate environmental fluctuations (Gehlot et al., 2022). Additionally, the cyanobacterial biomass generated through this process can be exploited as a feedstock for the production of a wide range of biobased products with several applications (Encarnação et al., 2023). However, the valorization of cyanobacterial biomass obtained after heavy metal bioremediation is still poorly investigated (Blanco-Vieites et al., 2023; Encarnação et al., 2023; Thevarajah et al., 2023).

In recent years the field of nanotechnology has shown increasing scientific and economic interest in the possible application of cyanobacterial biomass (Selmani et al., 2022). Nanotechnology-based on phototrophic microorganisms is called phyconanotechnology (Chan et al., 2022; Pandey et al., 2022b), and represents an opportunity for cyanobacterial biomass valorization. Nevertheless, research studies in cyanoremediation and phyconanotechnology are still unlinked.

Considering that cyanobacteria may represent a useful biotechnological tool to promote societal transitions toward a circular economy, through waste recovery and valorization, this article aims to illustrate recent trends and future directions in cyanobacteria-based heavy metal bioremediation and recovery of the metals and/or the valorization of the biomasses with a particular focus on phyconanotechnology. The combination of both processes will be suggested within a circular concept to ensure higher economic and environmental feasibility.

## 2. Heavy metal cyanoremediation: bioaccumulation vs. biosorption

Cyanobacteria exploit a variety of mechanisms to sequester and minimize the effect of heavy metals in contaminated environments, such as biosorption, bioaccumulation, and biotransformation. Metal-binding metallothionein (MT) proteins and phytochelatins (PCs), enzymatic and non-enzymatic antioxidants, and enzymes reducing heavy metals to less harmful forms play a pivotal role in cyanobacteria defense against heavy metals (Chakdar et al., 2022). A key role has been attributed to cyanobacteria exopolysaccharides (EPS), which are heteropolymers characterized by unique properties compared to other bacteria, including strong anionic nature due to the presence of one or two uronic acids and sulfate-containing sugars, and the presence of six or more different types of monosaccharides. The role of EPS in metal sequestration is mainly due to the presence of negatively charged groups, such as sulfate, phosphate, carboxyl, and hydroxyl, that may work as chelating agents for positively charged heavy metals (De Philippis and Micheletti, 2017; Cui et al., 2021; Potnis et al., 2021; Bhatt et al., 2022) and it has been widely demonstrated through molecular and biophysical techniques: mutagenesis, X-ray spectroscopy, Fourier transformed infrared spectroscopy (FTIR), and Scanning or Transmission Electron Microscopy (SEM–TEM) (Potnis et al., 2021).

Heavy metal remediation by cyanobacteria can be carried out through two main processes: bioaccumulation and biosorption (Bloch and Ghosh, 2022). The former is a metabolically driven active process that requires living cells, whereas the latter is a passive process that can be performed by both dead or living cells (Pandey et al., 2022a).

Biosorption, which is considered the major mechanism for the removal of heavy metals from wastewater, involves several mechanisms, such as ion exchange, adsorption, surface complexation, precipitation, and chelation (Bhatt et al., 2022). Since the cell wall of cyanobacteria is generally rich in negatively charged groups, which represent potential binding sites for heavy metals (De Philippis and Micheletti, 2017; Mota et al., 2022), in the first stage, metal ions can be rapidly sorbed to the surface of the cells. Consequently, metal ions can be translocated inside the cells through active transporters and carriers which are converted into less toxic forms and/or stored in vacuoles.

Several cyanobacteria genera, such as *Anabaena*, *Cyanobium*, *Nostoc*, *Cyanothece*, *Arthrospira*, *Microcystis*, *Synechocystis*, and *Leptolyngbya*, have shown promising results on Cu, Cd, Zn, Cr, Pb, Ni, Co or Hg removal with initial concentration ranging from some mg/L to 150–200 mg/L (Mota et al., 2016; Zinicovscaia et al., 2018; Yadav et al., 2021; Bloch and Ghosh, 2022; Pandey et al., 2022a). Maximum uptake is typically in the range of 15–80 mg/g dry weight, but some works have presented values even higher than 300 mg/g dry weight (Cui et al., 2021). Also, the use of consortia of different cyanobacteria species or microalgae/other microbes and cyanobacteria may help to attain higher metal tolerance as well as higher metabolite synthesis, positively contributing to metal removal (Cui et al., 2021). Nevertheless, the stability of the consortia should be monitored to ensure constant bioactivity. Generally, since the removal efficiency is maximized with a lower initial metal concentration, biosorption or bioaccumulation by cyanobacteria can be adopted after conventional methods that are characterized by low efficiency at a low heavy metal concentration (Agarwal et al., 2020).

Biosorption is considered a more feasible approach for heavy metal removal from wastewater compared to bioaccumulation, as it is characterized by faster kinetics, and the cells are not affected by heavy metal concentration which may be toxic at a high value. Nevertheless, several parameters are known to influence the biosorption process, such as pH, temperature, biosorbent dosage, and pretreatment, which require attention for improving the adsorption ability (Al-Amin et al., 2021). Another advantage of biosorption is represented by the possibility to exploit the cells for several desorption/adsorption cycles increasing their shelf-life and thus their economic value. During desorption, several solutions may be adopted, including strong acid or base, EDTA, or water, depending on the strength of the binding between metal ions and binding groups as well as the mechanical and physical strength of the biosorbents (Chatterjee and Abraham, 2019; Agarwal et al., 2020; Satya et al., 2021). Once eluted, the metal ions can be recovered to enter again inside the productive cycle of the industries, while the metal-free biomass can be used in a further adsorption cycle. When the adsorption capacity of the biomass is exhausted, the cells can be harvested for the last heavy metal recovery or their valorization.

It is worth mentioning cyanobacterial biomass management options. If cyanobacteria can be directly grown in open ponds or closed photobioreactors containing heavy metals contaminated wastewater exploiting active removal processes, other approaches should be implemented for biosorption since it doesn’t require metabolically active cells. The biomass obtained after their cultivation can be confined in closed systems with low porosity, such as dialysis membrane devices, or immobilized in polymeric matrices or filter-columns or filter-press (De Philippis et al., 2011; Ramírez Calderón et al., 2020). These systems are also advantageous to carry out adsorption/desorption cycles and/or for the recovery of metal-contaminated biomass that can be disposed for the following valorization. Since the materials remain confined or entrapped for the entire duration of the process, no risks due to biomass or metal contamination exist. Batch systems, which consist of metal-containing solutions and biosorbents, are commonly used in lab trials due to their simplicity. Nevertheless, the use of continuous reactors (e.g., fixed-bed column with continuous liquid flow) is favored at the industrial scale (Ramírez Calderón et al., 2020). Immobilization of cyanobacteria as biofilms has been recently developed as a feasible cultivation strategy to reduce water use and simplify the harvesting process (Cui et al., 2021). Immobilized cells or EPS onto a suitable carrier can be also used for the metal biosorption process, due to the increase in mechanical strength and chemical resistance of the biosorbents. Thus, multiple adsorption/desorption cycles can be easily carried out, and the exhausted biosorbents can be harvested at the end of the process for their valorization. In this context, adsorption, covalent bonds in vector compounds such as silica gel, entrapment/encapsulation in polymeric matrices, and cross-linking, can be exploited for immobilization. Nevertheless, the diffusion rate of metal ions into the polymeric matrices should be carefully checked (Velkova et al., 2018; Ajao et al., 2020; Cui et al., 2021). *Nostoc muscorum* immobilized on a glass surface through the formation of biofilm has been used for Cd removal from water solutions. The cultures exhibited higher cell resistance compared to the cell suspension and higher Cd tolerance (Raghavan et al., 2020). Velu et al. (2020) cultivated *Tolypothrix* sp. in outdoor cultures in simulated ash dam wastewater adopting 500 L vertical bag photobioreactor and as biofilms in algal-turf scrubbers. They found similar metal removal efficiency between the two cultivation systems.

Despite cyanobacteria biosorption has been widely recognized as an effective, fast, low-cost, and eco-friendly treatment method due to the large surface-to-volume ratio, the strong anionic character of EPS, and the possibility to regenerate, reuse, and easily recover the biosorbents (De Philippis and Micheletti, 2017; Singh, 2020; Priya et al., 2022), there are still economic and technical concerns that need to be managed for the process optimization, such as the economic cost and environmental footprint of biomass production, and metal recovery and reuse, that will be further faced in the following sections.

## 3. Heavy metal recovery and valorization

During the last decades, the necessity to minimize resource overexploitation and maximize waste prevention while generating economic gains has developed the circular economy concept (Velenturf and Purnell, 2021). This topic has been addressed by many research areas, including phycology (i.e., the study of algae). Many recent works investigated the cultivation of microalgae and cyanobacteria in conjunction with nutrient recycling from agro-industrial wastewater for biomass production and valorization (Abinandan et al., 2018; Gorain et al., 2019; Bhatt et al., 2022). However, research studies on heavy metal removal coupled with biomass application are still missing.

Cyanobacteria are commonly considered a potential source of bio-control agents, bio-fertilizers, soil amendments, food supplements, biofuels, high-value products, and biopolymers (Gomes Gradíssimo et al., 2020; Mona et al., 2020; Kholssi et al., 2021). Thus, the cyanobacteria biomass obtained after biosorption and/or bioaccumulation of heavy metal can be harvested and potentially converted into various economically significant by-products. Additionally, heavy metals can be recovered through desorption from metal-enriched biomasses or can be immobilized on biomass-derived carbons as metal-loading materials (Chai et al., 2022).

For instance, Serrà et al. (2020) proposed a circular zero-residue process adopting living cells of *Arthrospira platensis* for heavy metal bioremediation and the generated biomass for the production of bioethanol, biogas, and Fenton-like catalysts adopted for the degradation of persistent organic pollutants. Besides, the remaining low-activity ashes were used for the preparation of an ash-based medium for microalgae cultivation.

A further potentially interesting application that may be coupled with heavy metal removal is suggested by a recent work that investigated the use of hydrogels composed of sulphated polysaccharides from red microalgae and enriched with Zn as antimicrobial wound-dressing materials (Netanel Liberman et al., 2021).

## 4. Phyconanotechnology: synthesis and application

The valorization of metallic-organic materials obtained through metal biosorption in nanotechnology may represent a significant opportunity to increase the economic value of cyanobacteria. Nanotechnology is an emerging field concerning the synthesis, characterization, and application of nanomaterials (1–100 nm size) characterized by high surface area-to-volume ratio enhancing their physico-chemical properties (Hamida et al., 2020). The global nanotechnology market was valued at USD 9.39 billion in 2021 and is expected to register a CAGR of 14.9% by 2030 with increased application in electronics, followed by the medical industry (Market Analysis Report, 2021).

Among nanotechnologies, metal nanoparticles (NPs) are applicable in several fields, including diagnostic, biosensing, imaging, antimicrobials, catalysis, electronics, optics, biofuel cells, anticancer, and drug delivery (Hamida et al., 2020). Nevertheless, conventional methods for NP synthesis are often expensive and produce toxic by-products. To avoid these drawbacks, eco-compatible systems for the production of NPs are challenging: intra- and extracellular green synthesis of NPs adopting biological systems, including algae and cyanobacteria are receiving increasing attention (Ijaz et al., 2022; Mandhata et al., 2022; Barciela et al., 2023).

Microbes, and in particular prokaryotic organisms, have been demonstrated to be effective nano-factories, thanks to their ability to accumulate and detoxify heavy metals and to the presence of a wide range of reductase enzymes, microbial cells are able to immobilize and reduce heavy metals, through a dose-dependent process (Mandhata et al., 2022). Metal NPs synthesis can be carried out through a metabolism-dependent or independent process, where proteins and polysaccharides work as reducing, stabilizing, and capping agents (Mandhata et al., 2022). Generally, metal ions are entrapped into the cell surface thanks to the presence of negatively charged groups in the biosorption process, where they are reduced by enzymes, proteins, lipids, and pigments. Metal ions can also enter into the cell through internal absorption, to be reduced intracellularly by enzymes (nitrate reductase, nitrogenase) and then stabilized.

The term phyconanotechnology is referred to nanotechnology based on biobased material produced or constituted by photosynthetic microorganisms (Chan et al., 2022; Pandey et al., 2022b). For example, polysaccharides from brown algae (e.g., alginate, fucoidan, and laminaran) can be used as reducing and stabilizing agents for eco-friendly synthesis of silver NPs with cytotoxicity and antibacterial activity (Yugay et al., 2020). Recent studies have been carried out to obtain Cu, Zn, Cd, Ti, Au, and Ag NPs of different sizes (5–266 nm) and shapes (mainly spherical) adopting cyanobacteria EPS, cells, or cellular extracts as summarized in Table 1. These metal NPs have been tested for their application in medical, antimicrobial, and bioremediation fields (Saran et al., 2017; Ebadi et al., 2019; Ismail et al., 2021; Hanna et al., 2022; Mandhata et al., 2022; Pandey et al., 2022b). Most studies are based on AuNPs and AgNPs biosynthesis (Table 1) due to their non-toxicity, bio-compatibility, and for their high-potential therapeutic applications (Aziz et al., 2021; Mandhata et al., 2022). Although therapeutic and anti-microbial applications are commonly studied, the use of bio-based metal NPs in the industrial sector needs deeper investigation.

**TABLE 1:** Metal nanoparticle synthesis by different cyanobacteria.

Cyanobacteria	Synthesized nanomaterials	Shape and size	Application/Activity	References
*Anabaena variabilis*	Ag NPs	spherical and oval, 26 nm	dye removal	Ismail et al., 2021
*Anabaena spiroides*	Au NPs	different shapes, <80 nm	antimicrobial activity	Mandhata et al., 2021
*Anabaena* sp. 66-2	Ag NPs	irregular, 24 nm	antibacterial activity	Patel et al., 2015
*Arthrospira platensis*	Au NPs	spherical, 14 nm	antioxidant and catalytic activity	Zayadi and Abu Bakar, 2020
*Arthrospira platensis*	CuO NPs	spherical, 15 nm	photocatalytic activity	Alsamhary et al., 2022
*Arthrospira platensis*	ZnO NPs	spherical, 30–55 nm	antibacterial and anticancer activity	El-Belely et al., 2021
*Arthrospira platensis*	Au NPs	spherical, 15–60 nm	antibacterial activity	Uma Suganya et al., 2015
*Arthrospira platensis*	Ag NPs	spherical and oval, 18 nm	dye removal	Ismail et al., 2021
*Cyanothece* sp.	Ag NPs	different shapes, 80–129 nm	curative effect on myocardial infarction	Younis et al., 2019
*Leptolyngbya* sp. WUC 59	Ag NPs	spherical, 20–35 nm	antibacterial activity	Singh et al., 2020
*Microchaete* sp. NCUU-342	Ag NPs	spherical, 60–80 nm	dye decolorization	Husain et al., 2019
*Nostoc cameum*	Ag NPs	spherical, 7–27 nm	antibacterial, cytotoxic and antihemolytic activity	El-Naggar et al., 2018
*Nostoc commune*	Ag NPs	spherical, 15–45 nm	antifungal and antibacterial activity	Morsy et al., 2014
*Nostoc* sp. EA03	ZnO NPs	star, 20–80 nm	antibacterial, antibiofilm and anticancer activity	Ebadi et al., 2019
*Oscillatoria limnetica*	Ag NPs	spherical, 5–26 nm	antibacterial and anticancer activity	Hamouda et al., 2019
*Oscillatoria sp.* NCCU-369	ZnO NPs	spherical, 40–130 nm	antioxidant and antibacterial activity	Asif et al., 2021
*Phormidium tenue* NTDM05	CdS NPs	spherical, 5 nm	biolabeling	MubarakAli et al., 2012
*Synechococcus* sp.	AgNP	spherical, 15–266 nm	Photocatalytic and antibacterial activity	Keskin et al., 2016
*Synechocystis* NCCU-370	TiO_2_ NPs	spherical, 73 nm	antibacterial, antifungal and antioxidant activity	Siddiqui et al., 2022
*Synechocystis* sp.	Ag NPs	spherical, 10–35 nm	antimicrobial and wound-healing activity	Younis et al., 2022
*Synechocystis* sp. 48-3	Ag NPs	irregular, 15 nm	antibacterial activity	Patel et al., 2015

For instance, the development of functional textiles adopting metal NPs has triggered the interest of the industrial sector in recent years. The incorporation of metal NPs provides textiles with antimicrobial, ultraviolet-resistance, self-cleaning capabilities, and flame-retardant properties. Polysaccharides can reduce and stabilize metal NPs and promote their adhesion to fabrics. In addition, polysaccharides can improve the properties of textiles due to their physico-chemical characteristics (Fernandes et al., 2022). In this frame, cyanobacterial EPS, with metal chelating properties and a high amount that can be synthesized, could constitute an interesting tool for NPs stabilization and functional textiles improvement.

Additionally, metal NPs offer numerous benefits as green catalysts due to their high reactivity, selectivity, low cost, and easy preparation, and the fact that they can be widely applied for the production of pharmaceuticals and some commodity chemicals (Sheldon and Woodley, 2018). For instance, simple Pd complexes can be heterogenized into red algae-derived polysaccharide supports to improve conversion rates in Suzuki cross-coupling reaction (Wolfson et al., 2018), while CuNPs can be stabilized in chitosan-based hydrogel and used as a catalyst for the synthesis of 1,2,3-triazoles (Souza et al., 2019). Sulfated polysaccharides of algal origin have been also used as bio-matrix and capping agent for BaFe_12_O_19_ NPs. These heterogeneous materials were characterized by high catalytic activity in the one-pot synthesis of 2-amino-4H-pyrans and pyrans annulated heterocyclic compounds, effective reusability, and antibacterial activity (Amirnejat et al., 2022). The differences in the catalytic effectiveness between commercial polysaccharides and extracted microbial exopolysaccharides are probably ascribable to their different composition, for example to the presence of peculiar monomers in the polysaccharidic backbone of microbial EPS (Sutherland, 1990).

According to Sheldon and Woodley (2018), new developments in the biobased economy will further enhance broader applications of biocatalysis. The study of microbial cells or soluble chelating EPS for obtaining metal-bearing biocatalysts can be a new opportunity for developing innovative and eco-sustainable high value industrial products even in a circular economy concept.

For example, microbial biomass and EPS can be recovered after metal biosorption and used as biocatalysts for many organic transformations. In this context, Gandolfi et al. (2022) successfully valorized the use of two EPS-producing bacterial strains, using their biomass after Cu biosorption as hybrid catalysts in the asymmetric boron addition on α,β-unsaturated chalcones for the synthesis of valuable pharmaceutical intermediates.

Therefore, as a perspective, since many cyanobacteria species are excellent EPS producers in terms of quality and quantity (Cruz et al., 2020; Morais et al., 2022), and their monosaccharidic composition is highly heterogeneous, their application in the field of green catalysis may represent a huge opportunity.

**FIGURE 1:**
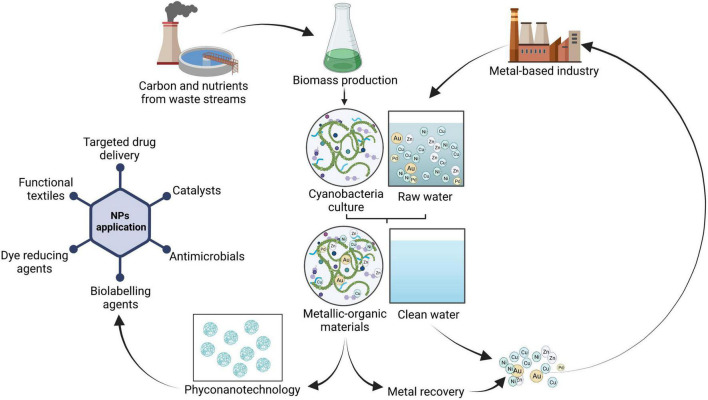
Schematic representation of heavy metal biosorption adopting cyanobacteria coupled with metal recovery and phyconanotechnology through a circular process. Created with BioRender.com.

Despite ongoing research in phyconanotechnology, the field is still at the beginning, this area of study is considered an option for increasing the market value and potential applications of EPS (Morais et al., 2022). Additionally, coupling heavy metal removal with metal NPs production may promote the development of a circular system to get high-added value products from waste as suggested in Figure 1. Nevertheless, new studies based on the elucidation of the mechanisms for biosynthesis of NPs and screening of different strains are needed to develop standardized protocols.

## 5. Future directions

Cyanobacteria advanced cultivation systems for higher target molecules and biomass productivity or improved downstream processing may be adopted to enhance the bioremoval process. Molecular engineering strategies targeting the ability, specificity, and robustness of cyanobacteria strains can also improve their properties. Even if general risks for cyanobacteria-based remediation are not illustrated in the literature, biosafety issues and contamination risk need to be considered for engineered cyanobacteria (Cui et al., 2021).

Another target to achieve is the reduction of the costs associated with biomass cultivation while increasing the environmental benefits of the process (Wan Mahari et al., 2022). Cyanobacteria can be cultivated by replacing conventional fertilizers with nutrient-rich wastewater (Figure 1), leading to a double outcome: the recovery of carbon, nitrogen, phosphorus, and other nutrients through their assimilation, together with cyanobacteria biomass production (Sachdeva et al., 2018; Gomes Gradíssimo et al., 2020; Kholssi et al., 2021; Prabha et al., 2022). Thus, the costs associated with artificial salts and water requirements are reduced together with the environmental footprint of the process (Sachdeva et al., 2018). The produced biomass can be harvested and pre-treated to be repeatedly used as biosorbent in multiple adsorption/desorption cycles for the continuous recovery of metals from wastewater. Moreover, when used at the end of the cycle, the obtained metal-organic materials can be valorized, exploiting the biochemical properties of cyanobacteria to achieve high-value products through phyconanotechnologies (Figure 1). In this context, the effect of biomass-specific properties, or metal type and quantity on the performance of the obtained materials needs to be explored together with their physical-chemical characterization. This approach may help to reach circularity and sustainability requirements, maximizing the valorization of wastes.

To date, a limited number of works have evaluated metal biosorption from industrial wastewater, despite their composition and pH being known to strongly influence the heavy metal removal process, including the selectivity of the biosorbent toward metals (Zinicovscaia et al., 2019; Li et al., 2022). Specific studies with metal-rich wastewater are needed to implement this approach at pilot and industrial scales. For instance, the recovery of water and metals from electroplating effluents is particularly challenging due to the high costs associated with the metal-coating process and the treatment of the generated effluents (Li et al., 2022). Thus, the cyanobacteria-based approach suggested in this article may be implemented to reduce metal concentration from these effluents, while obtaining a metal-rich organic material.

The environmental and economic benefits as well as the potential risks of converting and recycling heavy metal-contaminated biomass into value-added materials should be carefully evaluated. The valorization of these materials (cells or EPS) in different application fields, including nanotechnology, encompasses several safety concerns, which hinder their applicability. The increasing worldwide production of NPs and their low size may lead to their release into the atmosphere, aquatic, and terrestrial environments, through aggregation or biological transportation, reaching long distances. Due to their small size, NPs can easily cross biological barriers reaching any organ in living beings. Their bioaccumulation has been associated with many toxic effects, including alteration of the immune system, oxidation stress, carcinogenesis, and DNA damage (Solano et al., 2021; Liu et al., 2022). Size, shape, surface charge, and agglomeration state define several toxicological aspects of metal NPs (Fernandes et al., 2022). The risk assessment of these materials should be taken into account, even more accurately if the synthesis is coupled with heavy metal bioremoval from wastewater. Risk assessment and management process must be evaluated through the identification of the physic-chemical properties of the material, hazard identification, and dose-response assessment (Solano et al., 2021).

## 6. Conclusion

Cyanobacteria represent a potential tool for heavy metal bioremediation exploiting biosorption or bioaccumulation processes. Cyanobacteria cultivation, harvesting, pre-treatment, and exploitation in heavy metal bioremoval can be manipulated to get efficient metal recovery together with a reduced environmental footprint and the economic cost of the process. Nevertheless, considering cyanobacteria exclusively for their use in the bioremediation sector is economically disadvantageous. The metal-rich biomass obtained through this system can be converted into high-value products, such as metal nanoparticles, to be valorized into pharmaceutical and industrial fields, exploiting a closed-loop system with no waste production. However, more studies are needed to optimize heavy metal bioremoval at an industrial scale and also to explore a feasible and safe valorization of the obtained materials.

## Data availability statement

The original contributions presented in this study are included in the article/supplementary material, further inquiries can be directed to the corresponding author/s.

## Author contributions

MC and AA developed the concept of the manuscript and have critically read, and advised on improvements concerning the science and general outline of the manuscript. MC wrote the initial draft. Both authors contributed to manuscript revision, read, and approved the submitted version.
